# Supplementary material for: Monitoring of Dabrafenib and Trametinib in Serum and Self-Sampled Capillary Blood in Patients with BRAFV600-Mutant Melanoma
Source: Cancers (Basel). 2022 Sep 20;14(19):4566. doi: 10.3390/cancers14194566 (PMC9558510; doi:10.3390/cancers14194566)
Supplement: Supplementary file 1 [file cancers-14-04566-s001.zip › cancers-1876590-supplementary.pdf]

Supplementary Material

Monitoring of Dabrafenib and Trametinib in serum and self-sampled capillary blood in patients with BRAFV600-mutant melanoma

Nora Isberner[1, *], Anja Gesierich[2], David Balakirouchenane[3, 4], Bastian Schilling[2], Fatemeh Aghai-Trommeschlaeger[1], Sebastian Zimmermann[5], Max Kurlbaum[6, 7], Alicja Puszkiel[3, 4, 8], Benoit Blanchet[3, 4], Hartwig Klinker[1], Oliver Scherf-Clavel[5]

1 Department of Internal Medicine II, University Hospital Würzburg, Oberdürrbacher Strasse 6, 97080 Würzburg, Germany

2 Department of Dermatology, Venerology and Allergology, University Hospital Würzburg, Josef-Schneider-Strasse 2, 97080 Würzburg, Germany

3 Department of Pharmacokinetics and Pharmacochemistry, Cochin Hospital, AP-HP, CARPEM, 75014 Paris, France

4 Université Paris Cité, Faculté de Pharmacie, CiTCoM, 8038 CNRS, Inserm U1268, Paris, France

5 Institute of Pharmacy and Food Chemistry, University of Würzburg, Am Hubland, 97074 Würzburg, Germany

6 Department of Internal Medicine I, University Hospital Würzburg, Oberdürrbacher Strasse 6, 97080 Würzburg, Germany

7 Core Unit Clinical Mass Spectrometry, University Hospital Würzburg, Oberdürrbacher Strasse 6, 97080 Würzburg, Germany

8 Université Paris Cité, Faculté de Pharmacie, Inserm UMR-S1144, Paris, France

* Correspondance: isberner_n@ukw.de

..

Supplementary Table S1. Characteristics of serum samples

Sample characteristic	No. of samples (patients)	%
**Total**		
**(Hydroxy-)Dabrafenib**	**278 (27)**	100
included samples	277	99.6
excluded samples < LLOQ[a]	1[b]	0.4
**Trametinib**	**266 (27)**	
included samples	265	99.6
excluded samples < LLOQ	1[c]	0.4
**Samples / patient, median (range)**		
**(Hydroxy-)Dabrafenib**	10 (1 – 22)	
**Trametinib**	8 (1 – 22)	
**(Hydroxy-)Dabrafenib**[d]		
150 mg q12h	270 (27)	97.5
steady state samples	267 (27)	98.9
0-5 h post-dose	105 (17)	39.3
5-10 h post-dose	27 (10)	10.1
10-14 h post-dose	101 (16)	37.8
> 14 h post-dose	34 (14)	12.7
non steady state samples	3 (3)	1.1
100 mg q12h	7 (2)	2.5
steady state samples	7 (2)	2.5
non steady state samples	0	
**Trametinib**[e]		
2 mg q24h	214 (24)	80.8
steady state samples	211 (24)	98.6
0-10 h post-dose	61 (14)	28.9
10-20 h post-dose	34 (7)	16.1
20-30 h post-dose	115 (19)	54.5
> 30 h post-dose	1 (1)	0.5
non steady state samples	3 (3)	1.4
1,5 mg q24h	22 (2)	8.3
steady state samples	22 (2)	8.3
0-10 h post-dose	19 (2)	86.4
10-20 post-dose	1 (1)	4.6
20-30 h post-dose	2 (1)	9.1
> 30 h post-dose	0	
non steady state samples	0	
1 mg q24h	28 (5)	11.0
steady state samples	28 (5)	11.0
0-10 h post-dose	17 (4)	60.7
10-20 post-dose	1 (1)	3.6
20-30 h post-dose	10 (3)	35.7
> 30 h post-dose	0	
non steady state samples	0	

a LLOQ, lower limit of quantification (6 ng/mL for dabrafenib, 10 ng/mL for hydroxy-dabrafenib, 2 ng/mL for trametinib)
b sample collected at 75 mg 12 h post-dose
c sample collected at 2 mg 30 min post-dose
d steady state assumed after continuous dosing for 14 days
e steady state assumed after continuous dosing for 14 days

Supplementary Table S2. Characteristics of VAMS samples

Sample characteristic	No. of samples (patients)	%
**Total**		
**Dabrafenib**	**169 (18)**	
at-home samples	83 (9)	49.1
clinic samples	86 (18)	50.9
included samples	169	100
excluded samples < LLOQ	0	0
excluded samples due to incorrect sampling	8	4.7
**Trametinib**	**158 (18)**	
at-home samples	76 (8)	48.1
clinic samples	82 (18)	51.9
included samples	158	100
excluded samples < LLOQ	0	0
excluded samples due to incorrect sampling	8	5.1
**Samples / patient, median (range)**	**6 (1 - 22)**	
**Dabrafenib**		
150 mg q12h		
steady state samples	145	
non steady state samples	4	
0-5 h post-dose	64	
5-10 h post-dose	34	
10-14 h post-dose	45	
> 14 h post-dose	7	
100 mg q12h		
steady state samples	16	
non steady state samples	0	
0.5 h post-dose	4	
5-10 h post-dose	5	
10-14 h post-dose	7	
**Trametinib**		
2 mg q24h		
steady state samples	116	
non steady state samples	0	
0-10 h post-dose	47	
10-20 h post-dose	41	
20-30 h post-dose	28	
1.5 mg q24h		
steady state samples	22	
non steady state samples	0	
0-10 h post-dose	17	
10-20 h post-dose	4	
20-30 h post-dose	1	
1 mg q24h		
steady state samples	16	
non steady state samples	4	
0-10 h post-dose	6	
10-20 h post-dose	11	
20-30 h post-dose	3	

Supplementary Table S3. Steady state dabrafenib serum concentrations of all samples across all individuals

Dosing	time interval post-dose	per patient mean steady state dabrafenib serum concentration [ng/mL]				number of patients contributing samples	number of samples*	samples per patient	
		median	mean	IQR	SD			median	range
100 mg q12h	0-5 h	307.50	307.50	137.50	194.45	2	2 (0)	1 per patient	
	10-14 h	13.99	13.99			1	5 (0)	all from 1 patient	
150 mg q12h	0-5 h	844.38	1070.05	703.67	585.44	17	105 (0)	5	1-18
	5-10 h	260.75	268.69	194.55	188.77	10	27 (0)	2	1-7
	10-14 h	45.03	58.00	24.95	40.26	16	101 (0)	4.5	1-18
	> 14h	39.45	70.78	40.66	109.68	14	34 (0)	1	1-7

* no sample was < LLOQ (6 ng/mL)

Supplementary Table S4. Steady state hydroxy-dabrafenib serum concentrations of all samples across all individuals

Dosing	time interval post-dose	per patient mean steady state hydroxy-dabrafenib serum concentration [ng/mL]				number of patients contributing samples	number of samples (n < LLOQ)	samples per patient	
		median	mean	IQR	SD			median	range
100 mg q12h	0-5 h	341.59	341.59	218.87	309.53	2	2 (0)	1 per patient	
	10-14 h	37.21	37.21			1	5 (0)	all from 1 patient	
150 mg q12h	0-5 h	973.74	1210.67	753.64	780.51	17	105 (0)	5	1-18
	5-10 h	387.57	533.49	194.88	554.28	10	27 (0)	2	1-7
	10-14 h	76.01	87.26	51.43	39.68	16	101 (1)	4.5	1-18
	> 14 h	59.63	166.26	32.94	400.91	14	34 (0)	1	1-7

Supplementary Table S5. Steady state trametinib serum concentrations of all samples across all individuals

Dosing	time interval post-dose	per patient mean steady state trametinib serum concentration [ng/mL]				number of patients contributing samples	number of samples (n < LLOQ)	samples per patient	
		median	mean	IQR	SD			median	range
1.0 mg q24h	0-10 h	10.75	10.96	3.33	4.06	4	17 (0)	3	1-10
	10-20 h	13.80	13.80			1	1 (0)	only 1 sample	
	20-30 h	5.89	6.06	0.56	0.58	3	10 (0)	2	1-7
1.5 mg q24h	0-10 h	13.07	13.07	2.87	4.06	2	19 (0)	9.5	1-18
	10-20 h	8.92	8.92			1	1 (0)	only 1 sample	
	20-30 h	6.94	6.94			1	2 (0)	all samples from 1 patient	
2 mg q24h	0-10 h	17.50	18.09	5.82	5.31	14	62 (1*)		
	10-20 h	12.57	12.79	3.03	2.34	7	34 (0)		
	20-30 h	11.19	10.75	2.40	2.13	19	115 (0)		

* sample was collected 30 min post dose

Supplementary Table S6. Observed concentrations of dabrafenib, hydroxy-dabrafenib and trametinib vs. covariates

Compound	Covariate	groups	serum C_{min} [ng/mL]				n	p-value
			median	IQR	mean	SD		
Dabrafenib	sex	male	39.3	24.3	36.5	15	9	0.174
		female	52.9	77.8	75.5	48	7	
	age above 65	no	43.5	19.6	54.9	37.3	9	1
		yes	42.9	21.1	51.9	41.8	7	
	BMI (kg/m²)	< 30	42.9	21.4	46.5	34.4	11	0.377
		>= 30	54	76	69.1	44.8	5	
	moderate CYP2C8 inhibitor	no	44.3	28.7	58.9	49	9	0.689
		yes	47.4	17.4	46.9	15	6	
	P-gp inhibitor	no	52.9	86.2	77.8	54.6	7	0.351
		yes	41.8	9.9	42.4	13.8	9	
	P-gp inducer	no	48	38.3	60.9	46.1	10	0.594
		yes	41.8	9.86	40.4	9.31	5	
	PPI	no	51.6	35.4	63.1	43.1	13	0.305
		yes	32.4	9.42	32.4	13.3	2	
Hydroxy-dabrafenib	sex	male	72.9	38.7	77.3	41	9	0.837
		female	68.3	37.5	86	44	7	
	age above 65	no	77.6	69.7	92.7	47.7	9	0.252
		yes	55.6	29.1	66.2	27	7	
	BMI (kg/m²)	< 30	72.9	35.9	73.6	32.6	11	0.583
		>= 30	68.3	86	97.6	56.5	5	
	moderate CYP2C8 inhibitor	no	78	46.2	89.4	45	9	0.864
		yes	76.6	57	86.3	38	6	
	P-gp inhibitor	no	78	53.8	100	44.9	7	0.351
		yes	70.2	28	77.3	34.4	9	
	P-gp inducer	no	90.9	62.6	101	44.1	10	0.099
		yes	56.6	19.5	62.1	14.3	5	
	PPI	no	78	65.7	93.1	41.1	13	0.171
		yes	53.7	16.5	53.7	23.3	2	
Trametinib	sex	male	11.2	2.51	10.6	1.61	13	0.692
		female	11.3	2.01	11.1	3.08	6	
	age above 65	no	11.3	1.6	10.6	1.7	12	0.865
		yes	10.9	2.88	10.9	2.81	7	
	BMI	< 30	11.4	1.2	11.2	2.18	12	0.219
		>= 30	9.79	2.28	9.93	1.84	7	
	P-gp inhibitor	no	11.3	2.86	10.9	2.52	11	0.657
		yes	10.9	1.85	10.5	1.59	8	
	P-gp inducer	no	11.7	0.95	11.5	1.81	11	0.027
		yes	9.68	2.56	9.3	2.03	7	
	PPI	no	11.5	2.58	10.8	2.43	14	0.574
		yes	10.9	1.1	10.7	1.22	4	

C_{min}, trough concentration; IQR, interquartile range; SD, standard deviation; BMI, body mass index; PPI, proton pump inhibitor

Supplementary Table S7. Co-medication of all patients stratified by potential to induce or inhibit CYP3A4 or CYP2C8

Drug	CYP3A4	Strength	CYP2C8	Strength	Reference*
**Strong CYP3A4 or CYP2C8 inhibitors / inducers**					
Thiamazole	inhibitor	strong	-	-	(1)
**Moderate CYP3A4 or CYP2C8 inhibitors / inducers**					
Amlodipine	inhibitor	unknown	inhibitor	moderate	(1)
Clindamycin	inhibitor	moderate	-	-	(1)
Fluvastatin	inhibitor	unknown	inhibitor	moderate	(1)
Irbesartan	inhibitor	unknown	inhibitor	moderate	(1)
Levothyroxine	-	-	inhibitor	moderate	(1)
Spironolactone	-	-	inhibitor	moderate	(1)
**Weak CYP3A4 or CYP2C8 inhibitors / inducers**					
Candesartan	-	-	inhibitor	weak	(1)
Fenofibrate	-	-	inhibitor	weak	(1)
**CYP3A4 or CYP2C8 inhibitors / inducers of unknown strength**					
Atrovastatin	-	-	inhibitor	unknown	(1)
Hydrocortisone	inducer	unknown	inducer	unknown	(1)
Methylprednisolone	inducer	unknown	inducer	unknown	(1)
Omeprazole	inhibitor/inducer	unknown	-	-	(1)
Prednisolone	inducer	unknown	-	-	(1)
Prednisone	inducer	unknown	inducer	unknwon	(1)
Simvastatin	-	-	inhibitor	unknown	(1)
**Comedication without known effects on CYP3A4 or CYP2C8 metabolism**					
Acetylcysteine	-	-	-	-	(1)
Acetylsalicylic acid	-	-	-	-	(1)
Allopurinol	-	-	-	-	(1)
Amiloride	-	-	-	-	(1)
Bendroflumethiazide	-	-	-	-	(1)
Bisoprolol	-	-	-	-	(1)
Calcium	-	-	-	-	(1)
Calcium citrate	-	-	-	-	(1)
Carvedilol	-	-	-	-	(1)
Ceftriaxone	-	-	-	-	(1)
Clonidine	-	-	-	-	(1)
Denosumab	-	-	-	-	(1)
Digitoxin	-	-	-	-	(1)
Duasterid	-	-	-	-	(1)
Edoxaban	-	-	-	-	(1)
Enalapril	-	-	-	-	(1)
Estradiol	-	-	-	-	(1)
Hydrochlorothiazide	-	-	-	-	(1)
Ibuprofen	-	-	-	-	(1)
Ipilimumab	-	-	-	-	(1)
Iron	-	-	-	-	(1)
Lamotrigine	-	-	-	-	(1)
Levocetiricin	-	-	-	-	(1)
Levomepromazine	-	-	-	-	(1)
Lorazepam	-	-	-	-	(1)
Magnesium	-	-	-	-	(1)
Metformin	-	-	-	-	(1)

Metoprolol	-	-	-	-	(1)
Moxonidine	-	-	-	-	(1)
Mycophenolate mofetil	-	-	-	-	(1)
Nivolumab	-	-	-	-	(1)
Olmesartan	-	-	-	-	(1)
Omega 3 fatty acids	-	-	-	-	(1)
Pantoprazole	-	-	-	-	(1)
Penicillin	-	-	-	-	(1)
Phenprocoumon	-	-	-	-	(1)
Pravastatin	-	-	-	-	(1)
Quetiapine	-	-	-	-	(1)
Ramipril	-	-	-	-	(1)
Rivaroxaban	-	-	-	-	(1)
Selenium	-	-	-	-	(1)
Sitagliptin	-	-	-	-	(1)
Tacrolimus	-	-	-	-	(1)
Tamsulosin	-	-	-	-	(1)
Tilidin	-	-	-	-	(1)
Tiotropium	-	-	-	-	(1)
Torasemide	-	-	-	-	(1)
Triameteren	-	-	-	-	(1)
Trimipramine	-	-	-	-	(1)
Valsartan	-	-	-	-	(1)
Venlafaxine	-	-	-	-	(1)
Vitamin D3	-	-	-	-	(1)
Vitamin E	-	-	-	-	(1)
Xipamide	-	-	-	-	(1)

* (1) www.drugbank.ca

Supplementary Table S8. Co-medication of all patients stratified by potential to induce or inhibit P-glycoprotein

Drug	Reference*
**P-glycoprotein inhibitors**	
Amlodipine	(1)
Atrovastatin	(1)
Bisoprolol	(1)
Candesartan	(1)
Carvedilol	(1)
Fluvastatin	(1)
Lamotrigine	(1)
Omeprazole	(1)
Simvastatin	(1)
Tacrolimus	(1,2,3)
Venlafaxine	(1)
**P-glycoprotein inducers**	
Acetylsalicylic acid	(1)
Hydrocortisone	(1)
Levothyroxine	(1)
Methylpredinsolone	(1)
Prednisolone	(1)
Spironolactone	(1)
**Co-medication without known effects on P-glycoprotein**	
Acetylcysteine	(1)
Allopurinol	(1)
Amiloride	(1)
Bendroflumethiazide	(1)
Calcium	(1)
Clindamycin	(1)
Clonidine	(1)
Denosumab	(1)
Digitoxin	(1)
Dutasterid	(1)
Edoxaban	(1)
Enalapril	(1)
Fenofibrate	(1)
Fluticasone	(1)
Formoterol	(1)
Hydrochlorothiazide	(1)
Ibuprofen	(1)
Ipilimumab	(1)
Irbesartan	(1)
Iron	(1)
Iron (II) glycine sulphate	(1)
Lacosamide	(1)
Levetiracetam	(1)
Levocetiricin	(1)
Levomepromazine	(1)
Lorazepam	(1)
Magnesium	(1)
Metformin	(1)
Metoprolol	(1)
Moxonidine	(1)

Mycophenolate mofetil	(1)
Nivolumab	(1)
Olmesartan	(1)
Omega 3 fatty acids	(1)
Pantoprazole	(1)
Penicillin	(1)
Phenprocoumon	(1)
Potassium	(1)
Potassium citrate	(1)
Pravastatin	(1)
Quetiapine	(1)
Ramipril	(1)
Rivaroxaban	(1)
Salmeterol	(1)
Selenium	(1)
Sitagliptin	(1)
Tamsulosin	(1)
Thiamazole	(1)
Tilidin	(1)
Tiotropium	(1)
Torasemide	(1)
Triamteren	(1)
Trimipramine	(1)
Valsartan	(1)
Vitamin D3	(1)
Vitamin E	(1)
Xipamide	(1)

* (1) www.drugbank.ca

(2) Saeki T, Ueda K, Tanigawara Y, Hori R, Komano T. Human P-glycoprotein transports cyclosporin A and FK506. J Biol Chem. 1993 Mar 25;268(9):6077-80. PMID: 7681059.

(3) Singh H, Agarwal V, Chaturvedi S, Misra DP, Jaiswal AK, Prasad N. Reciprocal Relationship Between HDAC2 and P-Glycoprotein/MRP-1 and Their Role in Steroid Resistance in Childhood Nephrotic Syndrome. Front Pharmacol. 2019 May 22;10:558. doi: 10.3389/fphar.2019.00558. PMID: 31191307; PMCID: PMC6540828

Supplementary Table S9. Error metrics for the individual predictions using empirical Bayesian estimates

Compound	MPE [%]	MAPE [%]	MRD
Dabrafenib	-5.45	23.68	1.34
Hydroxy-Dabrafenib	26.76	43.8	1.60
Trametinib	3.9	15.5	1.23

Supplementary Table S10. Summarized MAP estimates for AUC$_\tau$ and trough concentrations of dabrafenib and hydroxy-dabrafenib

ID	pause / dose reduction / discontinuation due to AE	average predicted DAB AUC$_\tau$ [ng ·h /mL] 150mg q12h	average predicted OH-DAB AUC$_\tau$ [ng ·h /mL] 150mg q12h	sum of predicted AUC$_\tau$	average predicted DAB C_{min} [ng/mL] 150mg q12h	average observed DAB C_{min} [ng/mL]	average predicted OH-DAB C_{min} [ng/mL] 150mg q12h	average observed OH-DAB C_{min} [ng/mL]	sum of observed C_{min} [ng/mL]
DT001		5296	5450	10746	29.10	28.7	62.00	50.61	79.31
DT002		7809	4090	11899	121.36	115.2	82.73	74.02	189.22
DT003		6002	6200	12202	37.30	41.4	78.30	78.98	120.38
DT004		5676	5750	11426	41.42	41.8	82.08	70.16	111.96
DT005		6240	17810	24050	51.85	-	510.00	-	
DT006		6478	8210	14688	54.00	54.0	156.00	141.56	195.56
DT007	p	10535	11930	22465	183.21	-	362.14	-	
DT008	d	4720	6040	10760	20.50	-	67.50	-	
DT009	r	4460	5040	9500	39.50	20.0	84.50	72.90	92.90
DT010		3610	4480	8090	14.12	-	36.55	-	
DT011	d	8304	5250	13554	120.63	154.7	110.13	102.80	257.50
DT012		5423	4370	9793	36.06	45.8	51.00	56.61	102.41
DT013		6974	7800	14774	63.25	-	137.50	-	
DT014		3145	2490	5635	50.69	23.0	46.10	37.19	60.19
DT015		6371	6800	13171	67.71	69.8	128.43	122.33	192.13
DT017		6027	6930	12957	40.64	-	101.73	-	
DT018		5944	5500	11444	42.00	-	78.59	-	
DT019		5709	5590	11299	46.04	44.3	92.05	55.16	99.46
DT020		5796	6820	12616	28.50	34.4	80.40	83.13	117.53
DT021	d	3245	6320	9565	10.50	5.5	62.50	15.36	20.86
DT022		5100	4180	9280	78.63	20.8	61.75	78.00	98.80
DT023		8331	7890	16221	119.69	129.6	197.69	170.93	300.53
DT024		6400	4330	10730	45.00	52.9	53.00	50.08	102.98
DT025		3608	5860	9468	30.00	-	97.00	-	
DT026	r	4999	6440	11439	32.00	14.0	88.57	37.21	51.21
DT027		6350	8540	14890	42.29	51.7	141.43	151.71	203.41
DT028		8566	9380	17946	298.00	-	468.00	-	

DAB, dabrafenib; OH-DAB, hydroxy-dabrafenib

Supplementary Table S11. Summarized MAP estimates for AUC$_\tau$ and trough concentrations of trametinib

ID	pause / dose reduction / discontinuation due to AE	predicted AUC$_\tau$ [ng ·h /mL] 2 mg q24h	predicted C_{min} [ng/mL] 2 mg q24h	average observed C_{min} [ng/mL]
DT001		313.09	10.51	10.1
DT002		358.36	12.66	12.6
DT003		287.59	9.53	11.2
DT004	r	326.00	11.51	11.8
DT005	d	546.25	20.17	-
DT006		334.62	11.67	11.9
DT007		331.39	11.54	-
DT008	d	326.05	11.36	9.2
DT009	r	342.83	12.02	11.9
DT010		300.6	10.34	9.1
DT011	d	442.00	15.76	15.9
DT012		228.19	7.27	7.2
DT013		450.43	16.60	-
DT014		325.51	11.37	10.7
DT015		337.89	11.81	11.8
DT017	r	399.45	14.43	6.7
DT018	r	252.2	8.43	7.7
DT019	r	335.72	11.53	6.9
DT020	r	339.07	11.90	5.6
DT021	d	327.27	11.37	11.2
DT022		343.38	12.04	12.1
DT023		349.24	11.79	11.3
DT024		247.34	8.06	6.2
DT025		371.33	13.00	-
DT026		303.18	9.09	9.7
DT027		304.02	10.39	11.7
DT028		251.78	8.35	-

Supplementary Table S12. Clinical adverse events documented at least once

	No. of patients (%)				
Max. CTCAE grade per patient	0	1	2	3	≥ 4
Abdominal pain	23 (85.2)	4 (14.8)			
Arthralgia	21 (77.8)	6 (22.2)			
Asthenia	25 (92.6)	1 (3.7)	1 (3.7)		
Bradycardia	26 (96.3)	1 (3.7)			
Cephalgia	24 (88.9)	3 (11.1)			
Constipation	25 (92.6)	2 (7.4)			
Cough	21 (77.8)	6 (22.2)			
Diarrhoea	21 (77.8)	5 (18.5)		1 (3.7)	
Dry skin	19 (70.4)	7 (25.9)	1 (3.7)		
Dysgeusia	26 (96.3)	1 (3.7)			
Dyspnea	20 (74.1)	7 (25.9)			
Edema	22 (81.5)	5 (18.5)			
Fatigue	20 (74.1)	5 (18.5)	2 (7.4)		
Flatulence	26 (96.3)	1 (3.7)			
Flu-like symptoms	17 (63.0)	9 (33.3)	1 (3.7)		
Folliculitis / cellulitis	25 (92.6)	2 (7.4)			
Hair loss	25 (92.6)	2 (7.4)			
Impaired vision	26 (96.3)	1 (3.7)			
Loss of appetite	22 (81.5)	5 (18.5)			
Muscle cramps	25 (92.6)	2 (7.4)			
Myalgia	17 (63.0)	10 (37.0)			
Nail dystrophy	25 (92.6)	2 (7.4)			
Nausea	20 (74.1)	6 (22.2)	1 (3.7)		
Neoplasms of the skin	24 (88.9)	3 (11.1)			
Paronchyia	26 (96.3)	1 (3.7)			
Parotitis	26 (96.3)	1 (3.7)			
Pruritus	20 (74.1)	6 (22.2)	1 (3.7)		
Pyrexia	22 (81.5)	4 (14.8)		1 (3.7)	
Reduced LVEF	21 (77.8)	5 (18.5)	1 (3.7)		
Skin rash	22 (81.5)	5 (18.5)			
Stomatitis	26 (96.3)	1 (3.7)			
Urinary tract infection	26 (96.3)		1 (3.7)		
Vertigo	25 (92.6)	2 (7.4)			
Vomiting	24 (88.9)	2 (7.4)	1 (3.7)		
Weight gain	25 (92.6)	1 (3.7)	1 (3.7)		
Weight loss	25 (92.6)	2 (7.4)			
Xerostomia	24 (88.9)	3 (11.1)			

CTCAE, Common Terminology Criteria for Adverse Events; LVEF, left ventricular ejection fraction

Supplementary Table S13. Laboratory adverse events documented at least once

	No. of patients (%)				
Max. CTCAE grade per patient	0	1	2	3	≥ 4
Alkaline phosphatase elevated	9 (33.3)	16 (59.3)	2 (7.4)		
ALT elevated	23 (85.2)	4 (14.8)			
Anemia	4 (14.8)	21 (77.8)	2 (7.4)		
AST elevated	20 (74.1)	6 (22.2)	1 (3.7)		
Bilirubin elevated	26 (96.3)	1 (3.7)			
Creatine phosphokinase elevated	7 (25.9)	11 (40.7)	5 (18.5)	4 (14.8)	
creatinine elevated	15 (55.6)	12 (44.4)			
Gamma glutamyl transferase elevated	21 (77.8)	3 (11.1)	1 (3.7)	2 (7.4)	
Hyponatremia*	20 (74.1)	6 (22.2)			
Leucopenia	12 (44.4)	12 (44.4)	1 (3.7)	2 (7.4)	
Lipase elevated	13 (48.1)	9 (33.3)		5 (18.5)	
Lymphopenia*	14 (51.9)	3 (11.1)	7 (25.9)	2 (7.4)	
Neutropenia	20 (74.1)	4 (14.8)	2 (7.4)	1 (3.7)	
Thrombocytopenia	25 (92.6)	2 (7.4)			

ALT, alanine aminotransferase; AST, aspartate aminotransferase

* data not available for one patient

Supplementary Table S14. Dose reductions and treatment discontinuations

	permanent treatment discontinuation n (%)		temporary treatment discontinuation n (%)		dose reduction n (%)	
	dabrafenib	trametinib	dabrafenib	trametinib	dabrafenib	trametinib
none	19 (70.4)	18 (66.7)	17 (63.0)	17 (63.0)	24 (88.9)	21 (77.8)
adverse events	2 (7.4)*	3 (11.1)*	3+3** (22.2)	4+4*** (29.6)	2+1 (11.1)	1+5 (22.2)
end of adjuvant therapy	3 (11.1)	3 (11.1)	-	-	-	-
other reason	3 (11.1)	3 (11.1)	4** (14.8)	2 (7.4)	-	-

* two patients had permanent treatment discontinuation of dabrafenib *and* trametinib, one patient only of trametinib

** one patient had temporary treatment discontinuation before *and* during the study

*** two patients additionally had temporary treatment discontinuations for other reasons

\+ refers to patients with temporary treatment discontinuations or dose reductions *prior* to observation in the study

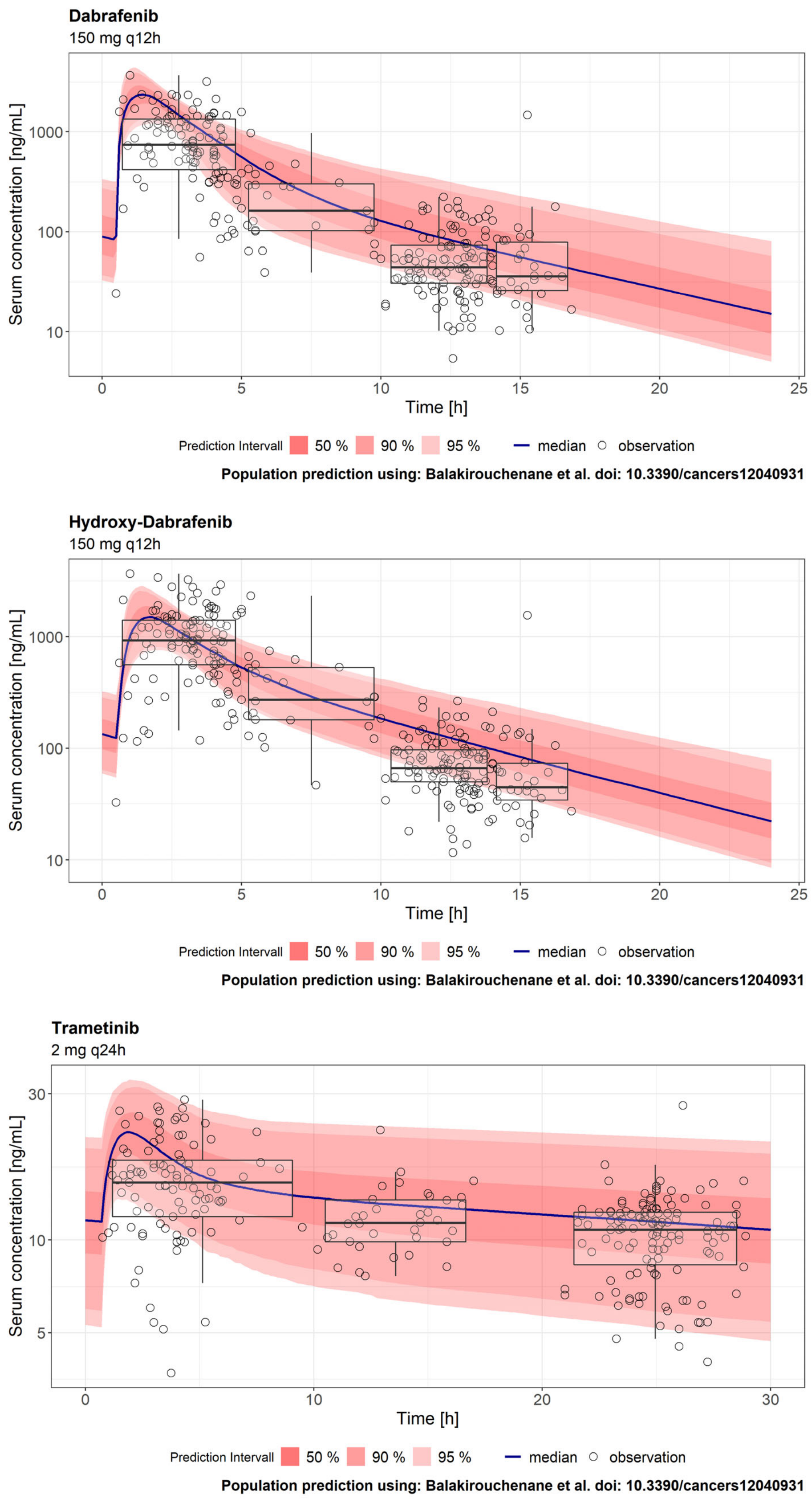

Supplementary Figure S1. Simulated steady state pharmacokinetics vs. observed data. Patient characteristics and dosages were used to simulate steady state pharmacokinetics with IIV on 1000 virtual patients. Simulated data was overlaid with the observed data.

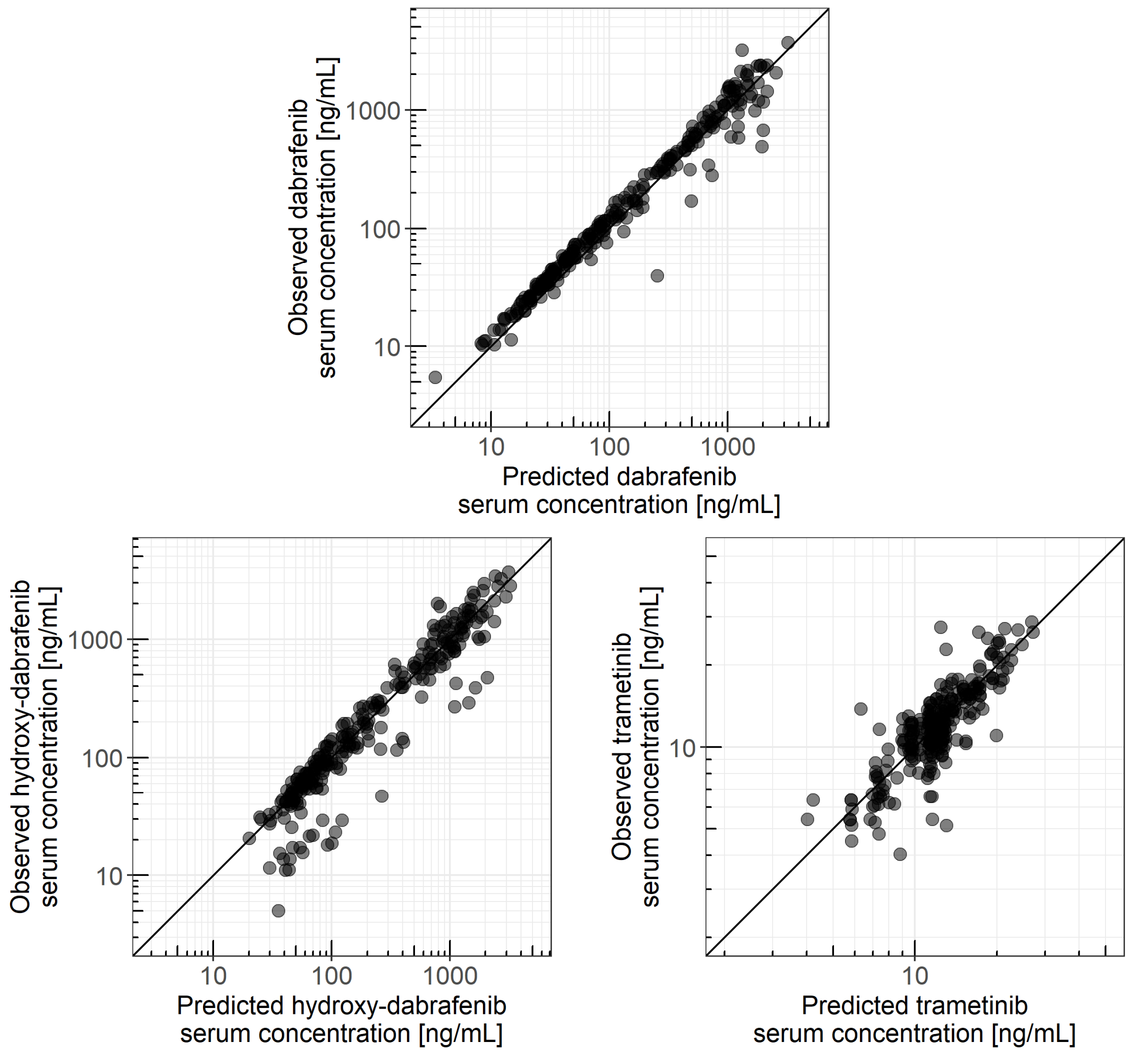

Supplementary Figure S2. Individual observed vs. individual predicted serum concentrations. Empirical Bayesian estimates were used for prediction of dabrafenib, hydroxy-dabrafenib and trametinib serum concentrations.

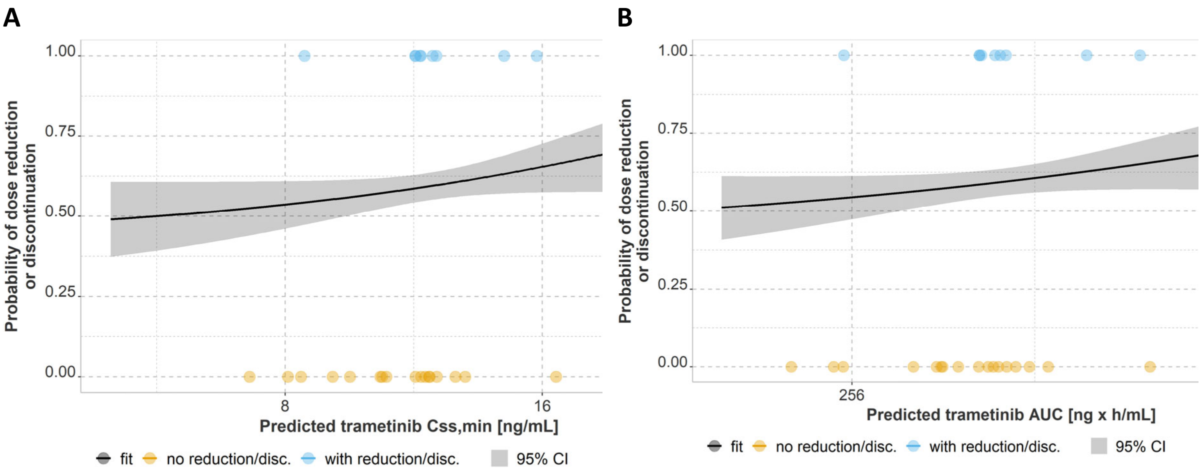

Supplementary Figure S3. Trametinib exposure vs. probability of dose reduction or treatment discontinuation. Logistic regression was used for analysis. **A:** Predicted trough concentration as exposure surrogate; intercept: -0.349, effect of C_{min}: 0.06178 (p-value: 0.0798) **B:** Predicted AUC_{τ} as exposure surrogate; intercept: -0.436, effect of AUC_{τ}: 0.0024 (p-value: 0.0988)

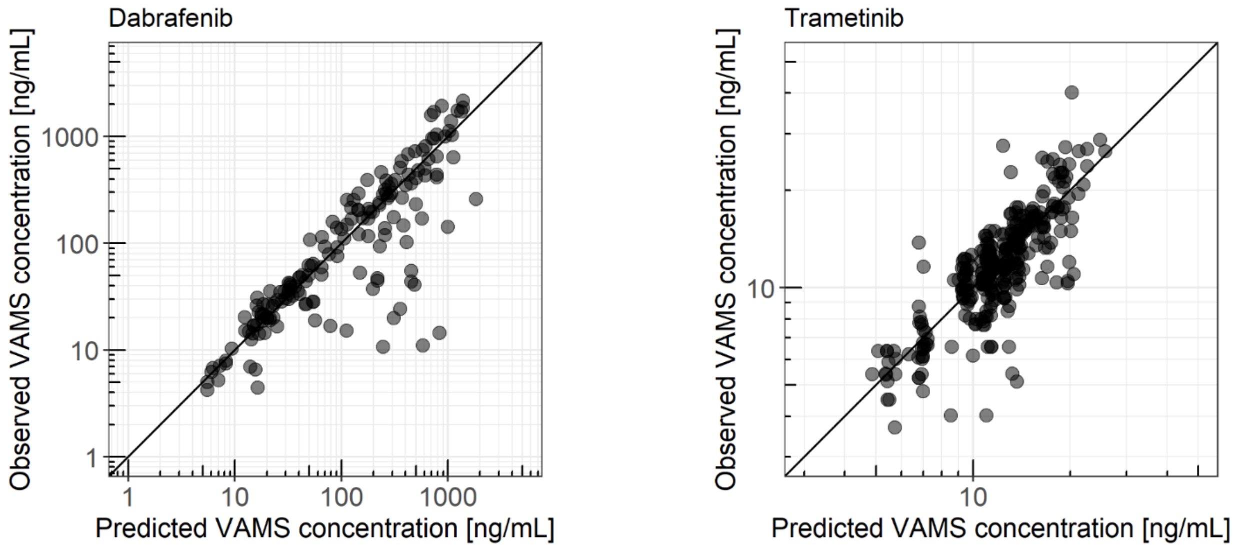

Supplementary Figure S4. Individual predicted vs. observed VAMS concentrations. Prediction of dabrafenib and trametinib concentrations was performed by using the consolidated datasets.

A

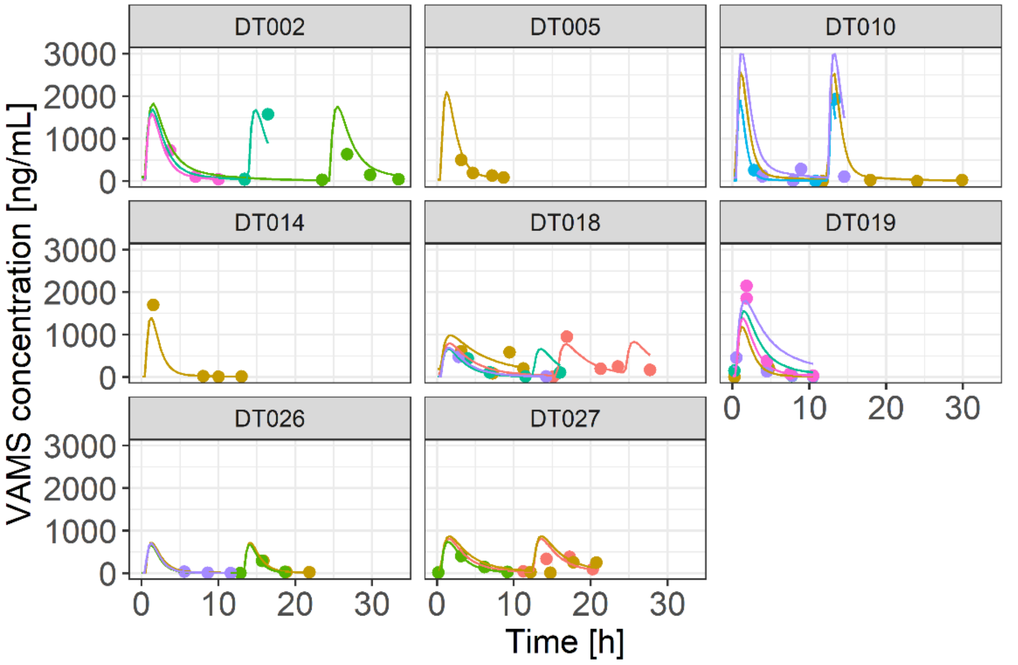

B

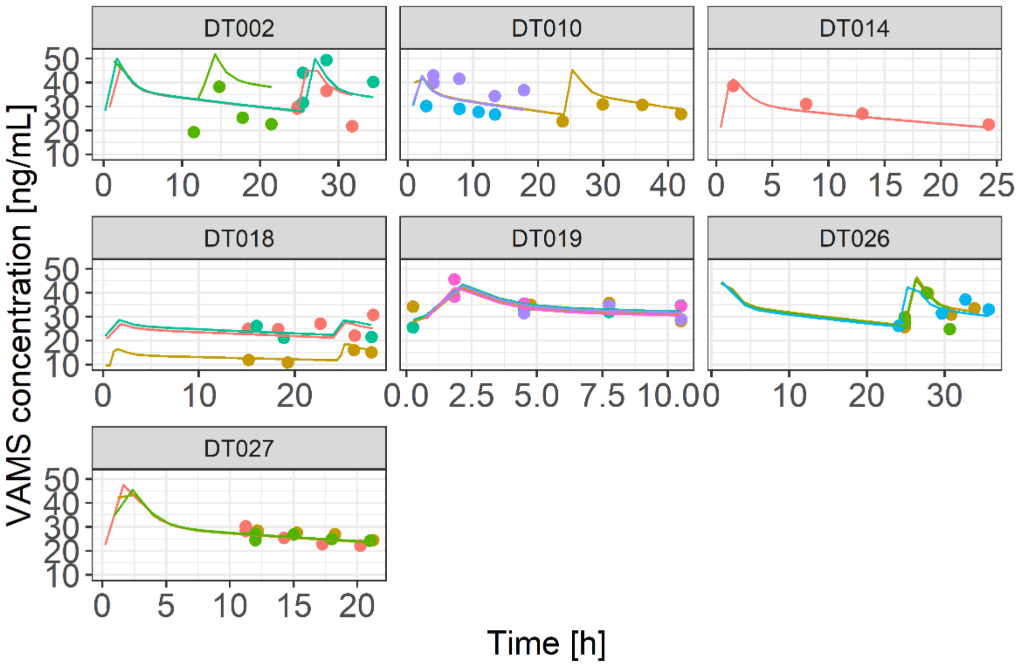

Supplementary Figure S5. At-home sampled VAMS concentration-time profiles. Points are individual measured VAMS concentrations; lines represent the fitted concentration time curve using the MAP approach. Different colours indicate different occasions of at-home sampling of the same patient. **A:** At-home sampled dabrafenib VAMS concentrations (90 samples, 8 patients). **B:** At-home sampled trametinib VAMS concentrations (84 samples, 7 patients). DT018 was not in steady state on one occasion. DT018 and DT019 received 1 mg and 1.5 mg q24h of trametinib, respectively. DT005 was on dabrafenib monotherapy.
